# PET/MR imaging for the evaluation of cervical cancer during pregnancy

**DOI:** 10.1186/s12884-021-03766-w

**Published:** 2021-04-10

**Authors:** Tatsuya Ishiguro, Nobumichi Nishikawa, Shiro Ishii, Kosuke Yoshihara, Kazufumi Haino, Masayuki Yamaguchi, Sosuke Adachi, Takafumi Watanabe, Shu Soeda, Takayuki Enomoto

**Affiliations:** 1 Department of Obstetrics and Gynecology, Niigata University Medical and Dental Hospital, 1-757, Asahimachi-dori, Chuo-ku, Niigata, 951-8510 Japan; 2 Department of Radiology and Nuclear Medicine, Fukushima Medical University Hospital, Fukushima, Japan; 3 Department of Obstetrics and Gynecology, Fukushima Medical University Hospital, Fukushima, Japan

**Keywords:** Cervical cancer, Pregnancy, PET-MRI, Metastasis

## Abstract

**Background:**

Malignancy during pregnancy is increasing, and the most common type of malignancy is uterine cervical cancer. When planning the treatment of cervical cancer, it is important to look for signs of metastasis before surgery, especially metastasis to the lymph nodes. In this report, we assessed the diagnostic value of positron emission tomography/magnetic resonance imaging (PET/MRI) for evaluating cervical cancer propagation before surgery, with a focus on pregnant women.

**Case presentation:**

^18^F Fluorodeoxyglucose **(**FDG)-PET/MRI was performed in seven pregnant cervical cancer patients (28–34 years old) at 9–18 gestational weeks. In case #5, a second PET/MRI was performed at 24 gestational weeks. Of seven FDG-PET/MRI examination series in six cases (cases #1–6), FDG-PET/MR imaging could detect cervical tumors with abnormal FDG accumulation; these tumors were confirmed with a standardized uptake value max (SUV max) titer of 4.5–16. A second PET/MRI examination in case #5 revealed the same SUV max titer as the first examination. In these six imaging series (cases #1–5), there were no signs of cancer metastasis to the parametrium and lymph nodes. However, in case #6, abnormal FDG accumulation in the left parametrial lymph nodes was also detectable. Pathological examination showed lymph node metastasis in case #6. In case #7, PET/MRI could not detect any abnormal FDG accumulation in the cervix and other sites. Cone biopsy demonstrated only micro-invasive squamous cell carcinoma. After treatment for cervical cancer, all seven patients have had no recurrence of disease within the follow-up period (2.8–5.6 years), and their children have developed appropriately.

**Conclusion:**

PET/MRI is an effective imaging tool to evaluate cervical cancer progression in pregnancy.

## Background

Uterine cervical cancer is one of the most common gynecological cancers. Since most cervical cancers develop as a result of persistent high-risk human papilloma virus (HPV) infection and multi-step carcinogenesis [1, 2], HPV vaccination and regular cancer screening, including HPV-DNA tests and Pap smears, are effective for the prevention of invasive cervical cancer in many women. Nevertheless, there are serious concerns related to cervical cancer in Japan. Firstly, the rate of the HPV vaccination of young women has dropped from 70% in 2013 to less than 1% in 2015 due to the Japanese Government’s suspension of proactive recommendations for the HPV vaccine due to suspected adverse events after HPV vaccination [3]. Secondly, the rate of women, especially young women, who undergo cervical cancer screening is extremely low compared with that in other developed countries [4]. Additionally, many younger women undergo a cervical cancer screening examination only at the initial prenatal examination in Japan. As a result, the disease rate of cervical cancer in Japan (13.3/100,000 women) is about the same as that in low- and middle-income countries (15.7/100,000 women) and higher than that in high-income countries (9.9/100,000 women) [1, 5].

Recently, malignancy during pregnancy has increased and the most common type of malignancy is uterine cervical cancer [6, 7]. Cervical cancer during pregnancy is mostly stage I disease, and the standard care of the International Federation of Gynecology and Obstetrics (FIGO) stage IB1 cervical cancer in pregnancy is radical hysterectomy with fetus in utero or cesarean radical hysterectomy [7]. Although it is most important to save maternal lives, patients occasionally wish to continue their pregnancy. To achieve the wish of patients without severe adverse effects on the fetus, we have previously reported the usefulness of abdominal radical trachelectomy during pregnancy (ART-DP) for stage IB1 cervical cancer [8]. Since pregnancy does not have a negative effect on cervical cancer prognosis, careful clinical and radiological follow up is another permissible treatment option for stage IB1 cervical cancer during pregnancy [7].

To plan the treatment, it is important to look for signs of metastasis before surgery. Cervical cancer progresses directly into the parametrium, vagina, uterus, and adjacent organs. In addition, it may progress further by spreading to the regional lymph nodes. Lymph node metastasis is one of the important risk factors of disease progression [9]. The incidence rate of pelvic lymph node metastasis with T-stage IA1, IA2, IB1, and IIA1 was reported as 1%, 0–4.8, 13.9, and 38.1%, respectively [10–12], and the rate of para-aortic lymph node metastasis with T-stage IB1 was 2–4% [13]. Therefore, the latest staging system of FIGO includes the lymph node metastasis status; cases with lymph node metastasis are diagnosed as stage IIIC [14].

To detect metastasis to the lymph node and parametrium before surgery, contrast-enhanced imaging including CT and MR are useful methods. However, the use of contrast-enhanced imaging during pregnancy, especially early gestation, must be avoided so as not to harm the fetus. Recently, fluorodeoxyglucose-positron emission tomography (FDG-PET) has been used to detect metastatic lesions of many types of cancer [15]. FDG-PET combined with CT or MRI has higher sensitivity than usual contrast-enhanced imaging for malignant tumors [16]. In this article, we discuss the usefulness of FDG-PET/MRI for cervical cancer treatment planning during pregnancy in seven cases of pregnant patients diagnosed with cervical cancer.

## Case presentation

Seven pregnant patients with cervical cancer were enrolled in this exploratory case series. They were treated at the Niigata University Medical and Dental Hospital, Niigata, Japan between 2013 and 2020. Abnormal cervical cells in all cases were first detected in early gestation by Pap smear screening, and malignant cervical tissues were subsequently confirmed by preoperative histological examination. The information related to these seven cases is summarized in Table 1. Of the seven cases presented in this study, three cases (cases #1–3) have been previously published [8].

**Table 1:** Description of the seven pregnant cervical cancer patients

Case	Stage	PET-MRI			Pathology					Treatment				Outcome	
		GA at PET-MRI, wk	SUV max		stage	histology	tumor size, mm	LVSI	pelvic LN metastasis	surgical procedure	GA at surgery, wk	GA at termination, wk	Adjuvant treatment	status	Period after surgery, y
			cervical cancer	pelvic LN											
#1	IB1	13 + 1	4.5	–	pT1b1N0	SCC	18	–	0/32	ART-DP	15 + 6	33 + 4	Cx	NED	5.6
#2	IB1	9 + 1	8.4	–	pT1b1N0	adenocarcinoma	22	–	0/15	ART-DP	15 + 1	37 + 1	–	NED	5.1
#3	IB1	15 + 6	6.6	–	pT1b1N0	SCC	15	–	0/24	ART-DP	17 + 0	33 + 0	–	NED	4.1
#4	IB1	13 + 0	10	–	pT1b1N0	SCC	20	–	0/30	ART-DP	15 + 4	37 + 3	–	NED	3.1
#5	IB1	18 + 3	6.4	–	pT1b1N0	SCC	30	ly (+)	0/22	C/S and ARH	31 + 4	31 + 4	Cx	NED	5.5
		24 + 2	7	–											
#6	IIIC	14 + 5	16	8	pT1b1N1	adenosquamous carcinoma	28	ly (+)	2/27	ARH	16 + 3	16 + 3	CCRT	NED	3.0
#7	IA1	14 + 5	–	–	pT1A1	SCC	2	–	–	conization	16 + 3	39 + 6	–	NED	2.8

*Abbreviations*: *GA* gestational age, *LN* lymph nodes, *SCC* squamous cell carcinoma, *LVSI* lymphovascular space invasion, *C/S* Cesarean section, *ART-DP* abdominal radical trachelectomy during pregnancy, *ARH* abdominal radical hysterectomy, *Cx* chemotherapy, *CCRT* concurrent chemoradiotherapy, *NED* no evidence of disease

^18^F-FDG- PET/MRI was performed at the Fukushima Medical University Hospital. PET/MRI data were acquired on an integrated PET/MRI system (Biograph mMR; Siemens Healthcare) with a 3.0-Tesla MRI. All patients fasted for at least 4 h or skipped one meal before the examination. The patients were injected with 4 MBq/kg of FDG, and PET/MR acquisition was started approximately 1 h after the FDG injection. The mean glucose level at the time of injection was 83.5 ± 6.5 mg/dl (range = 78–97). No contrast agent was used in any of the patients.

Acquisition of the PET data started from the upper thigh, progressing upwards to the head, under shallow breathing. MRI was acquired simultaneously with PET acquisition. The PET data were acquired for 4–6 bed positions with 3 min per bed position for the whole-body, followed by 10 min for the pelvis with a matrix of 172 × 172, and reconstructed using OSEM 3D.

Axial T2WI-HASTE (half-Fourier acquisition single-shot turbo spin echo), coronal T1WI (Turbo spin echo coronal T1-weighted images) from the top of the head to the upper thigh, and lung volumetric interpolated breath-hold examinations (VIBE) were obtained for the screening of metastases. In addition, 3-mm thickness of sagittal and axial T2WI (turbo spin echo T2WI) and T1-weighted images, axial DWI, and 3-mm thickness of pelvic MRI were obtained for evaluation of the invasion of cervical cancer.

**Fig. 1:**
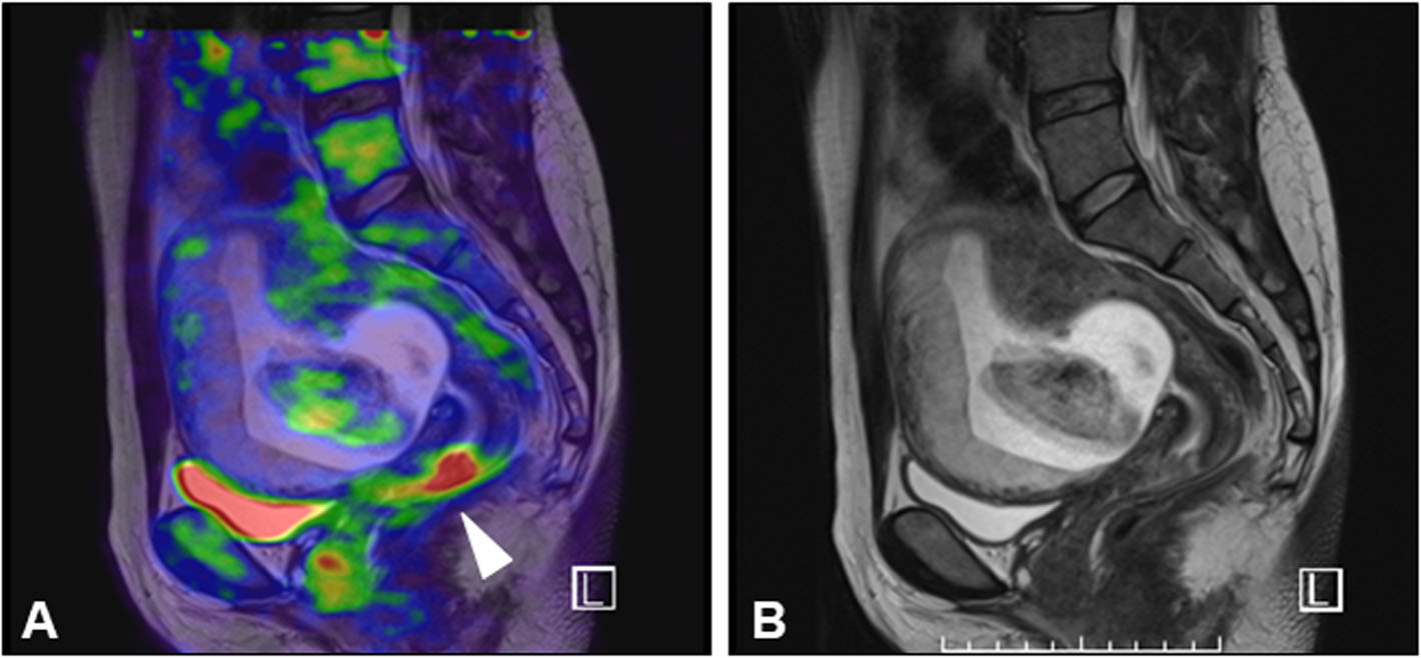
PET/MR images of case #3. **a** Sagittal view of PET/MR fusion image shows increased FDG uptake of cervical tumor (white arrowhead). **b** T2-weighted magnetic resonance image corresponding to PET/MR image (**a**)

**Fig. 2:**
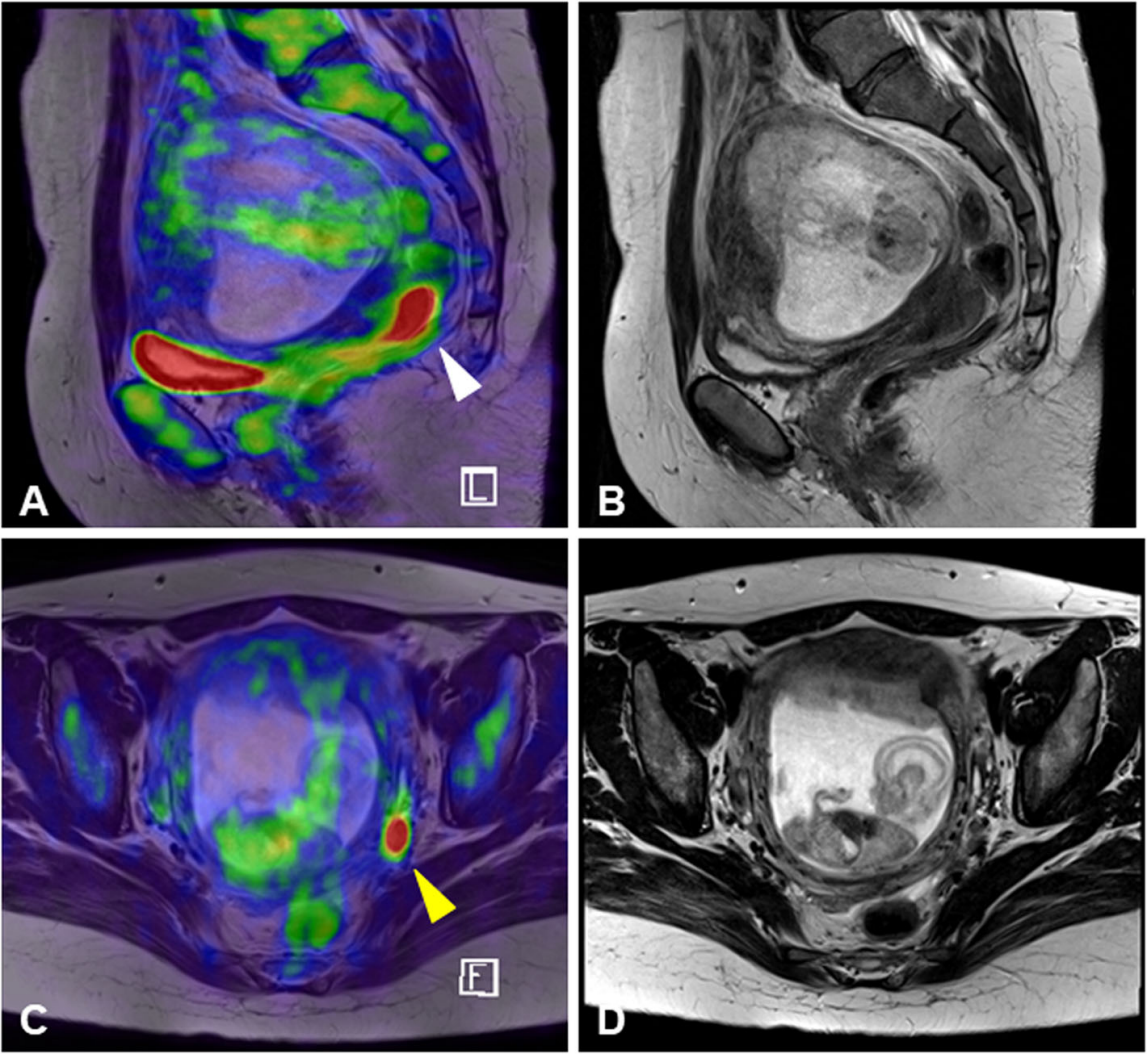
PET/MR images of case #6. **a** Sagittal views of PET/MR fusion image show increased FDG uptake of cervical tumor (white arrowhead). **b** T2-weighted magnetic resonance image corresponding to PET/MR image (**a**). **c** Axial views of PET/MR fusion image show increased FDG uptake of pelvic lymph node (yellow arrowhead). **d** T2-weighted magnetic resonance image corresponding to PET/MR image (**c**)

FDG-PET/MR imaging was performed for the seven pregnant patients (28–34 years old) at 9–18 gestational weeks in order to assess cervical cancer and metastatic lesions, including regional lymph nodes and the parametrium (Table 1). FDG-PET/MR imaging could detect cervical cancer with abnormal FDG accumulation in six FDG-PET/MRI examination series of the first five cases (cases #1–5); these tumors were confirmed with a standardized uptake value max (SUV max) titer of 4.5–10 (Fig. 1). In case #5, because uterine fibroid obstructed the ART-DP, FDG-PET/MRI was performed again at 24 gestational weeks to evaluate whether the patient could continue her pregnancy without any medical treatment for cervical cancer. The second FDG-PET/MRI examination showed the enhanced cervical tumor at the same SUV max titer as the first examination. In these six imaging series (cases #1–5), there were no signs of cancer metastasis to the parametrium and lymph nodes., In case #6, however, abnormal signaling of the left parametrial lymph nodes with an SUV max of 8 was found. This signaling was accompanied by FDG accumulation in the cervical tumor (SUV max of 16). However, MR imaging without enhanced contrast could not definitively detect the lymph node metastasis (Fig. 2).

The first surgery, including pelvic lymphadenectomy, was performed 8 days to 7 weeks after FDG-PET/MRI examination. More than 15 lymph nodes were removed during the surgery in all six cases (cases #1–6). Pathological examination showed the final diagnoses of squamous cell carcinoma (cases #1, #3, #4, #5, and #7), adenocarcinoma (case #2), and adenosquamous carcinoma (case #6). In addition, surgical exploration found no evidence of metastasis in the five cases that were found to be without metastatic lesion by FDG-PET/MRI (cases #1–5); in case #6 where lymph node metastasis was detected by FDG-PET/MRI, pathological examination also mainly revealed left obturator and parametrial lymph nodes metastases of squamous cell carcinoma. In case #7, the first punch biopsy sample of the uterine cervix revealed invasive carcinoma. Otherwise, PET/MRI could not detect any abnormal accumulation in the cervix or other sites. Cone biopsy demonstrated only micro-invasive squamous cell carcinoma with 2-mm deep stromal invasion.

Cesarean section after ART-DP was performed at 33 or 37 gestational weeks (cases #1–4). Simple hysterectomy was also performed at the same time as cesarean section; pathological cancer remnants were not detected in all four cases. In case #5, radical cesarean hysterectomy was performed at 31 gestational weeks. After cesarean delivery, two patients (cases #1 and #5) received adjuvant chemotherapy with paclitaxel and carboplatin because of deep stromal invasion (case #1) or the involvement of lymphatic space of cancer cells (case #5). Case #6, who received abdominal radical hysterectomy at 16 gestational weeks, received adjuvant concurrent chemo-radiotherapy with weekly cisplatin. After treatment for cervical cancer, all seven patients have had no recurrence within the follow-up period (2.8–5.6 years), and their children have developed appropriately.

## Discussion and conclusion

In this report, we described the usefulness of PET/MRI for the preoperative evaluation of cervical cancer metastasis during pregnancy. The guidelines for the management of cervical cancer patients from the European Society of Gynaecological Oncology/European Society for Radiotherapy and Oncology/European Society of Pathology recommend MRI and expert ultrasound examination followed by histological verification of cervical cancer involvement of suspicious lymph nodes to evaluate the cancer progression in pregnant patients [17]. Histological verification of nodes is an invasive procedure for pregnant patients, thus we consider that PET/MRI is more advantageous and can be used instead of this procedure.

Recently, it has been reported that FDG-PET imaging combined with CT or MRI are important tools to detect the spread of many cancers. Some groups have reported the efficacy of FDG-PET examination for cervical cancer [16, 18–20]. In a meta-analysis, the sensitivity and specificity of PET for lymph node metastases were 74.7% (95% CI 63.3–84.0) and 97.6% (95% CI 95.4–98.9), respectively. In this report, the sensitivity and specificity of MRI were reported to be 55.5% (95% CI 49.2–61.7) and 93.2% (95% CI 91.4–94.0), respectively and those of CT were 57.5% (95% CI 53.5–61.4), and 92.3% (95% CI 91.1–93.5), respectively [16]. In another retrospective analysis, the sensitivity, specificity, and false-negative and false-positive rates of PET/CT for detecting involved nodes were 53, 75, 6, and 82%, respectively [18]. In a prospective study of 18 gynecological cancer cases including cervical cancer and high-risk endometrial cancer, PET/MRI detected all primary tumors and regional lymph nodes as well as PET/CT. In addition, PET/MRI had higher sensitivity for parametrial and bladder invasion than PET/CT [19]. In another prospective study of 26 gynecological cancer cases including seven cervical cancer cases and 12 ovarian cancer cases, PET/MRI and PET/CT could equally detect regional lymph nodes metastasis [20]. In this report, the authors also reported that PET/MRI could detect parametrial invasion and invasion of the upper third of vagina more precisely than PET/CT. Based on these reports, PET/MRI is more effective in evaluating cervical cancer progression than other imaging techniques. Additionally, our detection of pelvic lymph node metastasis before surgery in case #6 using PET/MRI further supports these reports. However, while the sensitivity of PET examination is relatively high, as described above, the size detection limits of this examination have not been described. In case #6, one of two metastatic lymph nodes could not be detected with preoperative PET/MRI; a tiny primary cervical tumor in case #7 was also not detectable. False-negative results, due to small tumor size, and false-positive results, due to infection and inflammation, are possible. The study results suggest that an evaluation on PET/MRI should be considered, on the closest day before surgery, before performing an ART-DP for cervical cancer patients. If PET/MR imaging shows an abnormal accumulation anywhere except the uterine cervix, then histological verification is necessary during surgery.

Previously, the safety of FDG-PET imaging during pregnancy for the fetus has been discussed. In pregnant rhesus monkey models using ^18^F-fluorothymidine, there is higher radionuclide uptake and longer retention in the fetal liver than in the maternal liver [21]. Zanotti-Fregonara et al. calculated the fetal radiation dose for administration of ^18^F-FDG using mathematic modeling for placental crossover and fetal uptake. They reported that the estimated fetal dose ranged from 5.2 mSv in early pregnancy to 1.4 mSv in late pregnancy after an administered activity of 200 MBq was delivered; these doses were below the threshold for deterministic effects [22]. The results of this study encouraged the use of FDG-PET examination during pregnancy. In our cases, no abnormality among the children was detected during the follow-up period.

There is another unresolved clinical problem related to FDG-PET imaging in pregnancy, especially with regard to the number of times that PET/MRI can be performed in pregnancy. In case #5, we performed PET/MRI twice during pregnancy, at 18 + 3 and 24 + 2 gestational weeks, to monitor the cancer progression. Although a single PET examination seems to be tolerable for the fetus, as described above [23], the safety of repeated PET examinations has not been reported in detail. Further examinations with dosimetric calculations are needed to clarify the safety of PET/MRI for a fetus.

Although there were a limited number of cases and a short follow-up period, we found that FDG-PET imaging is a useful examination tool for the evaluation of cervical cancer before surgery. However, a large-scale study and further examinations are needed to resolve some concerns.

## Data Availability

Of the seven cases presented in this study, three cases (cases #1–3) have been previously published [8]. Other all data analyzed during this study are included in this report.

## Abbreviations

FDG: Fluorodeoxyglucose
PET: Positron emission tomography
MRI: Magnetic resonance imaging
SUV: Standardized uptake value
